# Integrating a Longitudinal Course on the Principles of Research in an Outcomes-Based Undergraduate Medical Education Curriculum

**DOI:** 10.5334/pme.1264

**Published:** 2024-10-14

**Authors:** Paul Farand, Tim Dubé, Marco Zaccagnini, Linda Bergeron, Justine Benoit-piau, Christina St-Onge

**Affiliations:** 1 Department of Medicine at Université de Sherbrooke, Sherbrooke, Québec, Canada; 2 Department of Family Medicine and Emergency Medicine at Université de Sherbrooke, Sherbrooke, Québec, Canada; 3 School of Epidemiology and Public Health, at the University of Ottawa, Ontario, Canada, and a respiratory therapist at the McGill University Health Centre, Montréal, Québec, Canada; 4 Faculty of Medicine and Health Sciences at Université de Sherbrooke, Sherbrooke, Québec, Canada; 5 Amsterdam Collaboration on Health & Safety in Sports, Vrije Universiteit Amsterdam, Amsterdam, Netherlands; 6 Department of Medicine at the Université de Sherbrooke, Sherbrooke, Québec, Canada

## Abstract

**Background and Need for Innovation:**

Teaching and learning approaches can support medical students in developing the research skills necessary to be adept consumers of scientific research. Despite various influencing factors, existing literature on effective strategies in undergraduate medical education remains limited.

**Goal of Innovation:**

Using a spiraled curriculum, we created and evaluated a longitudinal course to enhance medical students’ research abilities.

**Steps Taken for Development and Implementation of Innovation:**

During a recent curriculum renewal at one medical school, a three-year longitudinal course on the principles of research was developed and implemented. The innovation of this course includes the sequential nature and deliberate redundancy of curriculum content, how new knowledge is linked to prior learning, and the progressive level of difficulty in knowledge application and skill development.

**Evaluation of Innovation:**

The authors analysed faculty members’ and students’ satisfaction and their perceptions of each session of the course using program evaluation data collected between 2019 and 2021. Both faculty members and students recognized the benefits of revisiting concepts and highlighted learning outcomes like improved synthesis of information, explaining findings to patients, and enhanced critical thinking.

**Critical Reflection:**

The adoption of a spiraled curriculum in undergraduate medical education offers a systematic approach for developing students’ research skills. The positive reception of this innovation underscores its potential to help future health professionals form a professional identity as adept researchers. However, its implications demand careful consideration and ongoing evaluation to ensure that the desired outcomes are sustained.

## Background & Need for Innovation

The ever-evolving nature of healthcare necessitates that health professionals continuously understand and apply evidence-based practice in their clinical work to enhance clinical decision-making, foster professional development, drive improvements in medical practice, and prevent potential negative impacts on patient care [1,2]. Traditionally, undergraduate medical education (UGME) curricula have relied on *ad hoc* methods such as book clubs, workshops, and seminars to teach students research and critical analysis skills [3,4,5,6]. However, UGME programs continue to struggle with effective teaching and learning strategies aimed at developing these fundamental research and critical analysis skills required for medical practice [7]. One promising instructional approach is to interweave and integrate research and critical analysis teaching and learning into a spiralled curriculum. A spiralled curriculum is an approach that focuses on revisiting and building upon foundational concepts over time rather than covering a fixed set of topics in a linear timeline [8,9]. There are few examples in the literature of UGME curricula underpinned by this approach, and these tend to concentrate on specific content areas like concussions, palliative care, or oral health [10,11]. Explicit educational approaches and early exposure to research skills training in a UGME program could help better prepare students for the demands of residency and medical practice for their engagement in evidence-based practice and clinical decision-making. Teaching medical students the principles of research is challenging, but it is also foundational for their future role as consumers, producers and enactors of scientific research [12].

## Goal of Innovation

Our aim was to design an innovative longitudinal course underpinned by a spiral curriculum to teach research skills to medical students. Subsequently we evaluated the perceived benefits from the perspective of students and faculty members involved.

## Steps taken for Development and Implementation of innovation

In 2017, the University of Sherbrooke underwent a comprehensive revamping of its UGME program. The lead author (PF) assumed responsibility for developing and implementing a course focused on research skills, critical thinking, and foundational knowledge of research. This initiative stemmed from the acknowledgment that these skills had not historically received sufficient attention in the curriculum. During the developmental phases, PF sought input from researchers and educational advisors. A central committee was established to supervise the process, ensuring ongoing monitoring of the program, and overseeing necessary adjustments.

The resulting long-term course on research principles accounted for 4% of the total training time for medical students. Underpinned by a spiralled curriculum approach, this curriculum renewal focused on the learning outcomes students must achieve by the end of their UGME training [8]. The innovation of this course includes the sequential nature and deliberate redundancy of curriculum content, how new knowledge is linked to prior learning, and the progressive level of difficulty in knowledge application and skill development [8,13]. Through an explicit longitudinal content integration, the program designers emphasized the applied knowledge and the appropriate competencies medical students should develop during their UGME training [8]. By the end of this longitudinal course, students should be able to interpret scientific results by understanding the processes of conducting research and collaborating on— and executing— the different steps of projects on research questions related to medical practice.

The course is divided into two semesters to develop students’ knowledge of research fundamentals and epidemiology, followed by three semesters where students apply their research knowledge and skills through team-based learning activities, lectures, and self-learning modules. In this approach, the curriculum involves students learning the foundations of epidemiology through self-directed learning modules, followed by a team-based activity where students are grouped together with a faculty member to work on a real-world medical scenario related to the same topic (e.g., looking at a study demonstrating the effect of tobacco on lung cancer). After working with these real-world scenarios, students apply their knowledge through supervised working sessions, presentations, and participating in ongoing research by collecting data related to the topic. Another application of the curriculum includes students developing a research question and proposal aligned with a structured questionnaire. They are taught how to administer a structured questionnaire to research participants using best practice principles and participate in data collection, analysis, and interpretation of a collective cohort study. Research projects generally pertain to epidemiology questions and methodologies. With supervision, students select the topics and design the data collection method (usually surveys) to be used by students in the following year. These strategies aim to promote student engagement and facilitate the progressive acquisition of knowledge by revisiting and linking new material to previous knowledge as per the features of a spiraled curriculum.

**Figure 1:**
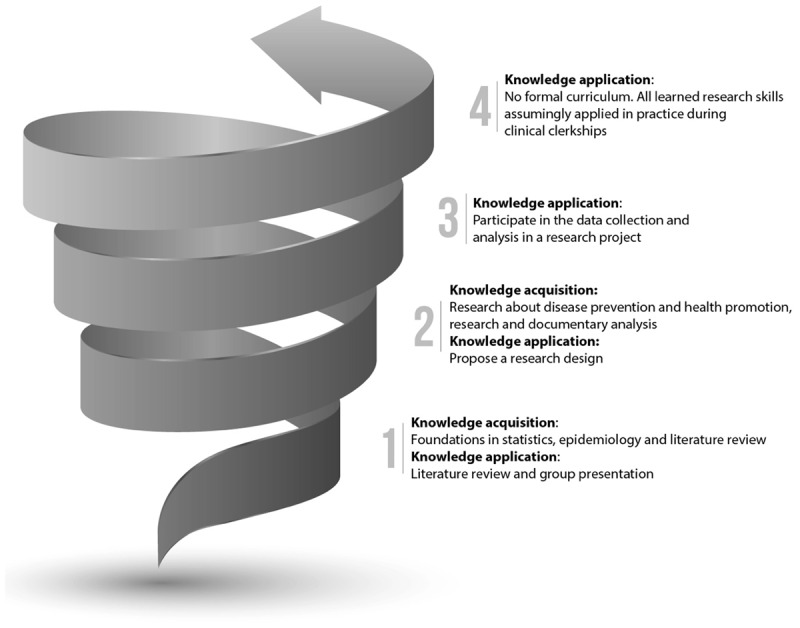
Four-year overview of the spiral curriculum for principles of research learning.

The course content is assessed using written exams (e.g., multiple choice questions, short answer questions, and extended-matching questions) for the knowledge acquisition components and rubrics for the knowledge application components. The rubric considers the expected evolution of a students’ performance in the dimensions corresponding to a competent act in a professional situation. Faculty members (i.e., instructors) use a rubric to write descriptive comments (e.g., strengths, challenges, and areas of concern) to support their assessment feedback to students and identify their demonstrated level of competence. This rubric is used to provide feedback to the student on the level achieved during the activity (low-stakes assessment) or at the end of the activity (high-stakes assessment). A snapshot of the curriculum is presented in Figure 1. A more detailed breakdown of the course, specific learning objectives, its structure and content are presented in Appendix 1.

## Evaluation of Innovation

We evaluated the self-perceived satisfaction and perceptions of the curriculum by analysing program evaluation data from UGME students and faculty members. This innovation was implemented in 2017, and we opted to use reports between 2019 and 2021 to allow for stability in the implementation of the renewed curriculum. Thus, program evaluation reports from each of the five semesters within the longitudinal course were provided to the research team by the UGME program. This project was exempt from ethical review by the research ethics board of the Université de Sherbrooke because the project used anonymized secondary data.

### Available data

The program designers created evaluation questions focused on how the course developed students’ research knowledge and skills. Students’ surveys contained between 15 and 17 items about the organisation of the course, the educational approach, the workload, satisfaction of the course, and appreciation of the faculty members. Faculty members’ surveys contained between 6 and 10 items about the material of the course, the standardisation of faculty members, the satisfaction of the course, and ease with the course content. Items were evaluated on a four-point scale (i.e., strongly disagree, somewhat disagree, somewhat agree, strongly agree) and contained a section for free-text comments. The program provided the research team with aggregated quantitative data and anonymized comments per semester.

For parsimony reasons, we present results from the evaluation questions that focused on the content and strategies specific to these courses. The remaining questions are general and are used indifferently in all course evaluations. We report means per question per stakeholder (faculty members and students) for each session. We conducted an adjunct analysis of the qualitative free-text comments on the surveys. We used Braun and Clarke’s thematic analysis with a deductive orientation to review these data [14]. Two team members familiarized themselves with the data (EF, LB). EF suggested an initial set of codes to LB who revised them. Codes were discussed with another member of the team (CSO), and the three began to generate themes. EF and LB met to define and describe the relevant themes.

### Results

The response rate for students varied between 13% (n = 28) and 48% (n = 104). The response rate for faculty members varied between 33% (n = 4) and 82% (n = 9). While the total number of students remained the same each semester, the number of faculty members varied per semester. We report in Appendix 2 the mean for each question per year and per semester, and provide illustrative quotes from the open comment section.

Both students and faculty members had a favorable perception of the course’s contribution to students’ development of knowledge and skills applied to medical research. Specifically, they favored learning about research methods, the literature review, the capacity to make concepts accessible, formulate a research question, collect, and analyse data, and interpret results.

A student explained that “*the course helps develop research skills useful for continuing education or future research endeavors, the accessibility of the content really helps to get a better idea of how to explain things to patients*” (MSP166_2019–2020). For some students, this course is a turning point and “*confirm[ed] my interest in research*” (MSP166_2020–2021). It helped them to develop “*critical thinking skills with regard to scientific articles*” (MSP305_2019–2020). Similarly, faculty members reported that they perceived the course had helped students develop the “*ability to synthesise information*” (MSP166_2021–2022).

They think that “*elaborating a research question, and then thinking of a methodology to answer it, is a strength for students*” (MSP266_2019–2020). Exposing students to a real research project is also very formative: “*the manipulation of data and the analysis of the results obtained improve their understanding of the different stages of a research project*.” (MSP305_2019).

In the comments, both students and faculty members expressed that the longitudinal aspect of the course and the deliberate and spiraled sequencing of each activity was progressive and logical to develop the appropriate competencies. Students said they “*liked the progressive learning of the steps of a research project*” (MSP166–2018–2019). The structure of the course allowed them to apply what they learn during the previous years (“*to apply the knowledge acquired during the winter [semester]*”, MSP166_2020–2021, MSP266–2018–2019). Students appreciate the intended revisiting of concepts: “*And it’s fun to practice what we did in practice last year. It’s definitely less difficult the second time around.”* (MSP266_2020–2021). Faculty members also expressed how the progression of knowledge and skills through the activities prepared students to apply their knowledge in a real research project: “*There is continuity: the progression of the three years brings the students to be well prepared for MSP305. Everything then becomes concrete in [the last semester] because of the opportunity to be involved in real activities (it is no longer just hypothetical as in the first two years.)*” (MSP305–2020–2021).

## Critical Reflection on the Process

We designed a longitudinal course in a UGME program to teach medical students research skills. Implementing the educational strategies in a spiralled curriculum allows reinforcement of the concepts to be learned, from simple to complex learning, with an integration of learning in a logical sequence [15]. This approach encourages students to go beyond just knowing research information; for example, medical students in our study reported applying their learning to professional situations [8].

Participating in this course can be a valuable experience for some students to develop an identity as a researcher. The course activities are composed of important elements to develop professional identities, such as mentoring, reflection, feedback, and role modeling [16]. The reflection component is particularly important as it enhances students’ critical thinking and decision-making by processing and applying their experiences to both scientific research and medical practice. Participating in this research course can help medical students develop their professional network by working with other students (e.g., medical, graduate) and faculty members. This can benefit students seeking opportunities to collaborate on future research projects or seeking advice or mentorship from more experienced researchers [17,18]. Additionally, the hands-on experiences with the research process (e.g., formulating a research question, administering a questionnaire, collecting and manipulating the data) can help students develop their skills and confidence as researchers. Each year, between 1,600 and 2,800 participants are included in the prospective cohort, with data collected by multiple students. Some findings have been published with students as co-authors, for example, Benoit-Piau et al. [19]. Finally, team-based learning, supervised working sessions, and low-stake assessments may all be strategies that support medical students in developing the appropriate competencies to undertake and/or critique scientific research.

This work has some limitations. It is a preliminary evaluation of a new curriculum using self-reported surveys. There might be a response bias, as students and faculty members who completed the questionnaires might have a stronger interest in the topic, which could affect the representativeness of the sample and the generalisability of the results. The response rate among students was relatively low and might be explained by the fact that students must complete an evaluation for each course, contributing to survey fatigue. There might have been other factors (e.g., personal or situational) that may affect students’ experiences with the course and their development as a researcher that could not be adequately captured by questionnaires.

The spiraled and longitudinal sequence of curricular activities proposed for teaching research skills seems promising. However, it’s essential to further evaluate its effectiveness among medical students and throughout residency training. Additional evaluation would help determine if the course is teaching the intended skills effectively and efficiently and might also aid in enhancing and refining the curriculum. This can be achieved in future empirical work by testing medical students pre- and post-course to determine any change in knowledge about research skills. Additionally, qualitative methodologies can be used to explore the notion of developing students’ identities as researchers after the course or to gain an in-depth understanding of students’ experiences in applying their knowledge and skills after UGME.

Finally, tracking students’ outcomes as they progress through their residency training would be valuable. For example, how many students end up publishing their completed research papers, or present their abstracts at conferences, and how many continue pursuing research into postgraduate residency could serve as important outcomes. Research productivity can be a surrogate to show how well students can apply the research skills they have learned in a real-world setting [20]. Additionally, tracking research productivity can provide insight into students’ ability to work independently and manage their time effectively. Conducting research often requires a high level of self-motivation and organization, and tracking productivity can help identify students who can effectively manage their workload and complete research projects [21].

## Appendix

**Appendix 1:** Details about the course, its structure and content.

COURSE	MSP105	MSP166	MSP205	MSP266	MSP305

**When**	Fall – Year 1	Winter-summer – Year 1	Fall – Year 2	Winter-summer – Year 2	Fall – Year 3

**Credits**	1 (15 hours)	2 (30 hours)	1 (15 hours)	2 (30 hours)	2 (30 hours)

**Objectives**	At the end of the educational activity, the student will be able to: ▪ interpret the results of scientific studies based on a simple design in epidemiology and in the evaluation of health prevention and promotion interventions; ▪ apply research skills to support the proposal of primary, secondary, and tertiary prevention activities in public health, and to conduct a literature review on a question related to medical practice.	At the end of the educational activity, the student will be able to: ▪ Conduct a literature review on a question related to medical practice: ▪ by relying on a literature search methodology; ▪ by undertaking a critical evaluation of the scientific literature related to the question; ▪ by collaborating, if necessary, with a literature review research team; ▪ by communicating the results of the literature review; ▪ by demonstrating commitment and professionalism towards the scientific community;	At the end of the educational activity, the student will be able to interpret the results of scientific studies based on a simple design in epidemiology and in the evaluation of health prevention and promotion interventions. Apply research skills to support the proposal of primary, secondary, and tertiary prevention activities in public health, and to conduct a literature review on a question related to medical practice.	At the end of the educational activity, the student will be able to: Propose a research design related to a question in medical practice: ▪ By choosing the most appropriate research design for the studied question; ▪ By conducting a critical evaluation of the possible research designs; ▪ By analyzing the impact of potential designs on the well-being and safety of subjects; ▪ By collaborating within one’s learning community; ▪ By presenting and defending one’s design to a peer group; ▪ By acknowledging one’s responsibilities in terms of research when developing a design;	At the end of the educational activity, the student will be able to participate in the data collection and analysis of a study related to a research question in medical practice: ▪ By relying on a rigorous research methodology appropriate for the objectives of the research project; ▪ By critically and systematically comparing the obtained results with those published in the literature; ▪ By prioritizing the well-being of subjects in activities involving them directly or indirectly; ▪ By collaborating with the research team; ▪ By communicating in writing the results of one’s project and analysis;

**Objectives**		▪ by recognizing ethical issues when conducting a literature review, and addressing them through reflection on ethical challenges and by identifying appropriate action strategies. Analyze one’s literature review practice.		▪ By recognizing ethical issues related to the respect of individuals and animals participating in research, when designing a research project, and addressing them through reflection on ethical challenges and by identifying appropriate action strategies. Analyze one’s research practice related to the development of a design.	▪ By demonstrating commitment and professionalism towards individuals and animals involved in the research; ▪ By recognizing ethical issues related to the respect of individuals and animals participating in the research, during the conduct of research, and addressing them through reflection on ethical challenges and by establishing appropriate action strategies. Analyze one’s research practice related to data collection and data analysis.

**Curriculum Content**	- Introduction to epidemiology. - Introduction to descriptive and inferential statistics. - Principle of the power of a study. - Steps of the scientific processes. - Types of designs and their characteristics. - Association calculations, incidence, and prevalence. - Biases and level of proof of studies. - Sensibility, specificity, positive and negative predictive value of a test, Hill’s criteria for evaluation of causality. - Definitions of research questions. - Use of different documentary sources and research engines. - Techniques to rapidly identify relevant resources.	- Identification and analysis of documentary resources to realize a literature review, analysis of own research practice. - Popularization and communication of the results of their literature review. - Concepts of plagiarism, intellectual property, veracity in documentation resources used, social responsibility in regards of data publication, and literature review rigor.	- Deepening of quantitative and qualitative research designs and systematic reviews, and their characteristics. - Choice of statistical tests. - Power of a design. - Critical appraisal of a study, including the biases that impact internal and external validity. - The notion of Number needed to treat/harm - Introduction to pharmaco-economy analysis of a study. - Notion of research patient and his rights; research ethics committee; research consent and ill patient vulnerability; animal as research subjects.	- Criteria to assess a research design and develop argumentation about the choice of a research design. - Communication techniques to create a positive environment for discussion of divergent opinions. - Recognize irritants in collaborative work. - Concepts of consent, confidentiality, and other aspects of participants safety related to the different research designs.	- Deepening of some biases and limits of some statistical test. - Process of data analyses. - Techniques of analyses and results communication. - Techniques of dissemination. - Principle of interdependency in collaboration, role clarification. - Concepts of consent, confidentiality, and other aspects of participants safety related to data collection and data analyses.

**Teaching and learning strategies**	- 4 team-based learning activities. - 1 lecture. - 2 self-learning modules - 1 practical session on literature review.	- 2 lectures. - 2 self-learning modules. - 2 supervised working sessions. - Students presentation of popularization of their results.	- 1 lecture. - 4 team-based learning activities. - 2 self-learning modules.	- 3 lectures. - 4 supervised working sessions. - 1 team-based learning activity.	- 1 lecture. - Students’ standardisation for survey administration. - 3 supervised working sessions. - Students participation in the design of the cohort study, to survey administration and to data analysis.

**Assessment methods**	30 questions divided in 2 exams. Around half of questions are multiple choice questions, 40% are short-answer questions and 2–4 questions are EMQ.	Low-stakes assessment at the beginning and high-stakes assessment at the end of the activity.	30 questions divided in 2 exams. Around half of questions are multiple choice questions, 40% are short-answer questions and 2–4 questions are EMQ.	Low-stakes assessment at the beginning and high-stakes assessment at the end of the activity.	Low-stakes assessment at the beginning and high-stakes assessment at the end of the activity.

EMQ = Extended Medical Questions.

**Appendix 2:** Mean score (/4) for questions related to the appreciation of the course per year and per course response for students and faculty members.

RESPONDENT TYPE	COURSE	QUESTIONS*	2019	2020	2021

Students	MSP105	This activity allows me to better understand the research methods related to medical practice.	3.1	3.2	3.0

		This activity helps me develop skills to interpret the results of clinical studies.	3.1	3.3	3.0

Faculty members	MSP105	This educational activity promotes the acquisition of knowledge in statistics and epidemiology related to medical practice.	3.5	4.0	3.5

		This educational activity promotes the acquisition of knowledge in the understanding and critical appraisal of medical literature.	3.3	3.8	3.5

Students	MSP166	The proposed activities allow me to apply my research skills.	3.6	3.6	3.5

		The proposed activities allow me to apply my presentation skills	3.7	3.6	3.4

Faculty members	MSP166	This activity promotes the acquisition of the essential skills for research and presentation related to medical practice.	3.3	4.0	3.5

		Presentations allow students to transfer the knowledge they have gained into the scientific and medical field.	3.5	4.0	3.7

Students	MSP205	This activity allows me to better understand the research methods related to medical practice.	3.5	3.7	3.2

		This activity helps me develop skills to interpret the results of clinical studies.	3.5	3.7	3.3

Faculty members	MSP205	This educational activity promotes the acquisition of knowledge in statistics and epidemiology related to medical practice.	3.9	3.8	3.7

		This educational activity promotes the acquisition of knowledge in the understanding and critical appraisal of medical literature.	3.6	3.8	3.7

Students	MSP266	The proposed activities allow me to apply my research skills.	3.7	3.6	3.8

		The proposed activities allow me to apply my skills to develop a research protocol.	3.8	3.7	3.8

Faculty members	MSP266	This educational activity helps students acquire the skills necessary to develop research specifications related to medical practice in relation to medical practice.	3.9	3.9	3.7

		The activity of designing research designs allow students to transfer the knowledge acquired to analyse and understand the literature in the scientific and medical practice.	3.7	4.0	3.8

Students	MSP305	The proposed activities allow me to apply my research skills.	3.2	3.4	3.5

		The activities allow me to participate in the data collection of a research project.	3.4	3.6	3.8

		The activities allow me to participate in the data analysis of a research project.	3.2	3.4	3.6

Faculty members	MSP305	The activity promotes the acquisition of skills necessary for research projects.	3.2	3.4	3.7

		The activity allows students to be involved in data collection for research.	3.5	3.5	3.9

		The activity allows students to be involved in the analysis of research data.	3.2	3.2	3.4

		The activity is useful to prepare future physicians.	3.4	3.7	3.8

		The activity is useful to help students analyze and understand scientific and medical literature.	3.2	3.2	3.4

*Translated from French.
